# Lipid metabolism of phenol-tolerant *Rhodococcus opacus* strains for lignin bioconversion

**DOI:** 10.1186/s13068-018-1337-z

**Published:** 2018-12-28

**Authors:** William R. Henson, Fong-Fu Hsu, Gautam Dantas, Tae Seok Moon, Marcus Foston

**Affiliations:** 1 Department of Energy, Environmental and Chemical Engineering, Washington University in St. Louis, St. Louis, MO 63130 USA; 2 Mass Spectrometry Resource, Division of Endocrinology, Diabetes, Metabolism, and Lipid Research, Department of Internal Medicine, Washington University School of Medicine, St. Louis, MO 63110 USA; 3 Department of Pathology and Immunology, Washington University in St. Louis School of Medicine, St. Louis, MO 63108 USA; 4 The Edison Family Center for Genome Sciences and Systems Biology, Washington University in St. Louis School of Medicine, St. Louis, MO 63110 USA; 5 Department of Biomedical Engineering, Washington University in St. Louis, St. Louis, MO 63130 USA; 6 Department of Molecular Microbiology, Washington University in St. Louis, St. Louis, MO 63108 USA

**Keywords:** *Rhodococcus opacus*, Phenol, Triacylglycerol, Mycolic acid, Phospholipid, Mass spectrometry

## Abstract

**Background:**

Lignin is a recalcitrant aromatic polymer that is a potential feedstock for renewable fuel and chemical production. *Rhodococcus opacus* PD630 is a promising strain for the biological upgrading of lignin due to its ability to tolerate and utilize lignin-derived aromatic compounds. To enhance its aromatic tolerance, we recently applied adaptive evolution using phenol as a sole carbon source and characterized a phenol-adapted *R. opacus* strain (evol40) and the wild-type (WT) strain by whole genome and RNA sequencing. While this effort increased our understanding of the aromatic tolerance, the tolerance mechanisms were not completely elucidated.

**Results:**

We hypothesize that the composition of lipids plays an important role in phenol tolerance. To test this hypothesis, we applied high-resolution mass spectrometry analysis to lipid samples obtained from the WT and evol40 strains grown in 1 g/L glucose (glucose), 0.75 g/L phenol (low phenol), or 1.5 g/L phenol (high phenol, evol40 only) as a sole carbon source. This analysis identified > 100 lipid species of mycolic acids, phosphatidylethanolamines (PEs), phosphatidylinositols (PIs), and triacylglycerols. In both strains, mycolic acids had fewer double bond numbers in phenol conditions than the glucose condition, and evol40 had significantly shorter mycolic acid chain lengths than the WT strain in phenol conditions. These results indicate that phenol adaptation affected mycolic acid membrane composition. In addition, the percentage of unsaturated phospholipids decreased for both strains in phenol conditions compared to the glucose condition. Moreover, the PI content increased for both strains in the low phenol condition compared to the glucose condition, and the PI content increased further for evol40 in the high phenol condition relative to the low phenol condition.

**Conclusions:**

This work represents the first comprehensive lipidomic study on the membrane of *R. opacus* grown using phenol as a sole carbon source. Our results suggest that the alteration of the mycolic acid and phospholipid membrane composition may be a strategy of *R. opacus* for phenol tolerance.

**Electronic supplementary material:**

The online version of this article (10.1186/s13068-018-1337-z) contains supplementary material, which is available to authorized users.

## Background

Lignocellulose is a potential source of renewable fuels, chemicals, and materials, but it requires efficient conversion for commercial viability [1–3]. Lignocellulose is composed primarily of cellulose, hemicellulose, and lignin [4]. Cellulose and hemicellulose can be easily depolymerized into sugars which can be converted via microbial fermentation to bioproducts [3, 5, 6]. However, lignin was designed by nature to give plants structural rigidity and to help plants resist biological attack, which makes industrial enzymatic or microbial depolymerization of lignin difficult [7, 8]. A hybrid approach to lignin conversion has recently been proposed, which combines the rapid depolymerization kinetics of catalytic or thermochemical processing with the metabolic funneling and selective bioproduction capabilities of microbial systems [9–11]. In this approach, lignin is thermochemically or catalytically depolymerized into diverse aromatic compounds, which are then converted by microbes into a single stream of valuable bioproducts [12]. However, lignin-derived aromatic compounds are toxic to most microbes and can reduce product titers, yields, and productivities in the fermentation of lignocellulosic hydrolysates [13]. Efforts are underway to improve the aromatic tolerance of microbes used for fermentation of lignocellulose-derived sugars and to develop strains that have aromatic degradation pathways to convert lignin-derived aromatics into valuable products [14–18].

*Rhodococcus opacus* PD630 (hereafter *R. opacus*) is an important microbial strain for bioproduction due to its inherently high aromatic tolerance and ability to consume many different aromatic compounds found in depolymerized lignin [19–24]. *R. opacus* is a Gram-positive actinomycete bacterium that can accumulate triacylglycerols (TAGs), a biodiesel precursor, up to ~ 78% of its cell dry weight when grown on sugars [25]. Moreover, with the newly developed synthetic biology tools [26–28], *R. opacus* could also be engineered for the bioconversion of lignin into many other bioproducts. While many studies have focused on characterizing the lipid metabolism of *R. opacus* for the goal of improving TAG accumulation [29–32], it is unknown whether, or how, the lipid metabolism plays a role in the tolerance of aromatic compounds.

Cell membrane structure and composition are important for microbial stress tolerance. The cell membrane acts as a permeability barrier for the influx and efflux of different compounds, and microbes respond to stressful growth conditions by modifying the structure of their cell membrane [33, 34]. For example, different yeast strains change their cell membrane composition under solvent stress [35, 36], acid stress [37], and freezing or salt stress [38]. For tolerance to aromatic compounds such as toluene, Gram-negative bacteria such as *Pseudomonas putida* convert *cis*-double bonds to *trans*-double bonds on fatty acid chains, increase amounts of cyclopropane fatty acids, and modify phospholipid head groups [39–41].

**Fig. 1:**
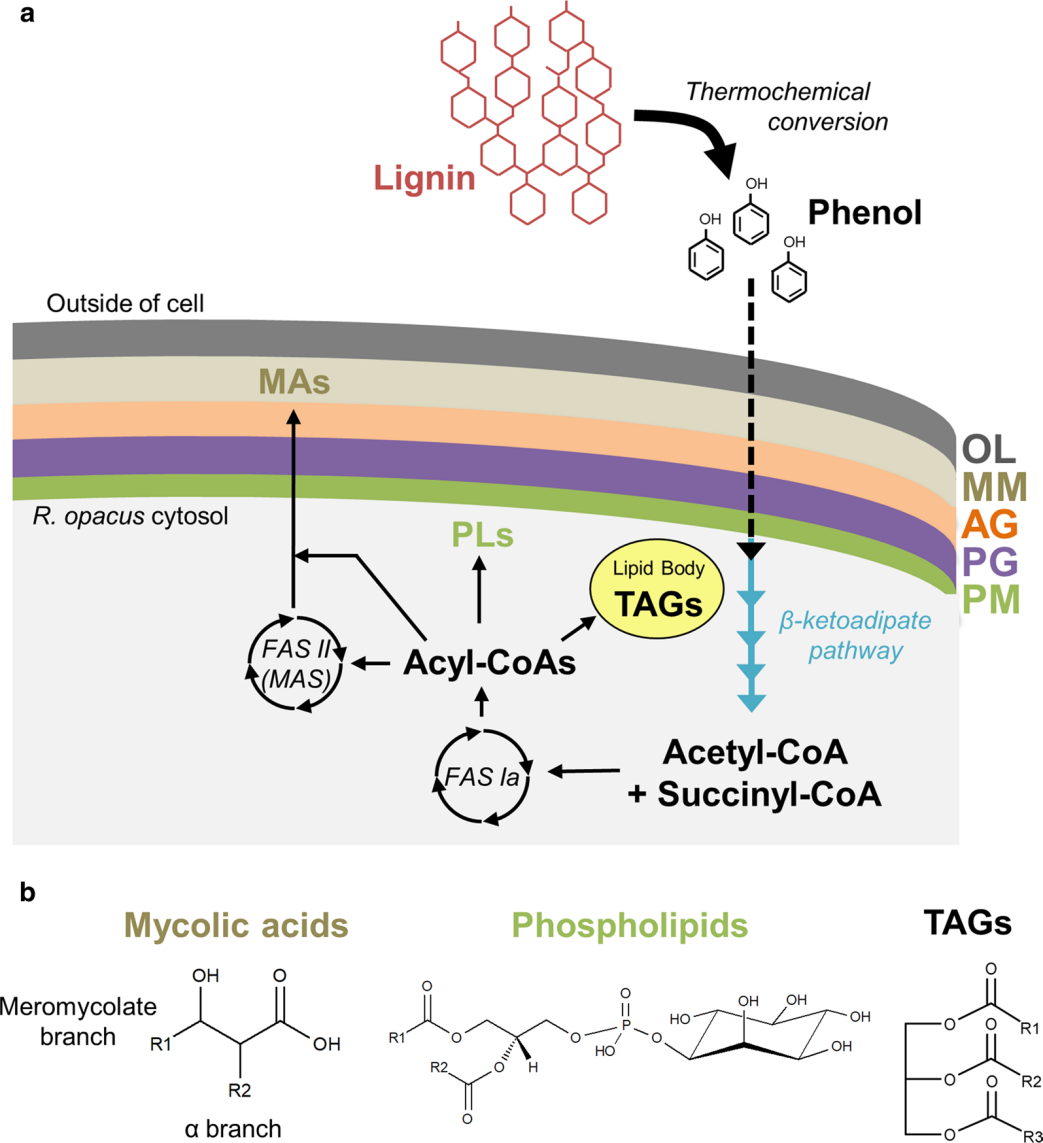
Conversion of phenol to lipids by *R. opacus* PD630. **a** Cell membrane structure and simplified model for conversion of a lignin monomer (phenol) to lipids by *R. opacus* PD630 [42]. Phenol is imported into the cell and converted to central metabolic intermediates acetyl-CoA and succinyl-CoA [20, 72]. Next, acetyl-CoA is converted into acyl-CoAs by fatty acid synthase (FAS) Ia [32]. Phospholipids (PLs), triacylglycerols (TAGs), and α-branches of mycolic acids (MAs) are synthesized using acyl-CoAs from FAS Ia, while meromycolate branches for mycolic acids are synthesized by further chain elongation using other fatty acid synthases (FAS II) or mycocerosic acid synthases (MAS) [32]. Triacylglycerols can be stored in lipid droplets [30]. *PM* phospholipid membrane, *PG* peptidoglycan layer, *AG* arabinogalactan layer, *MM* mycomembrane, and *OL* outer layer. **b** Representative structures of lipid types examined in this work.

*Rhodococcus* belongs to the Mycolata taxon that includes *Corynebacterium*, *Mycobacterium*, and *Nocardia* [42]. These bacteria have an unusually complex cell envelope compared to other Gram-positive bacteria (Fig. 1). For example, *R. opacus* has an outer membrane of mycolic acids (mycomembrane) [43–45] in addition to a phospholipid membrane [46, 47]. Studies in *Rhodococcus* strains indicate that they decrease fatty acid chain lengths and increase the amount of branched chain fatty acids during growth using aromatic compounds [48, 49], and the presence of aromatic compounds has been shown to alter the composition of meromycolate chains of mycolic acid species [50–52]. However, changes in intact lipid species using soft ionization techniques have not been extensively studied in *R. opacus* grown using aromatic compounds as a sole carbon source, and their role in aromatic tolerance remains to be elucidated.

Mass spectrometry is a useful tool for measuring changes in lipid composition in response to aromatic compounds. Collision-induced dissociation (CID) multiple-stage high-resolution mass spectrometry (HR-MS) provides detailed structural information which is essential for the identification of lipid species in non-model microbes [50]. In this work, we employed HR-MS together with liquid chromatography/mass spectrometry (LC/MS) to characterize cell membrane lipids, including mycolic acids and phospholipids, and biofuel target lipids, TAGs [23, 53]. Our approach enables the determination of the total carbon number and double bond number that each intact lipid molecule has, while the approach using fatty acid methyl ester formation, followed by gas chromatography/mass spectrometry (GC/MS) [32, 49], can provide only information regarding whole cell fatty acid compositions rather than intact lipid compositions. In this work, the total carbon number is defined as the number of carbons present on all three acyl chains of each TAG molecule, both acyl chains of each phospholipid molecule, and the meromycolate and alpha branches of each mycolic acid molecule. Similarly, the total double bond (DB) number is defined as the number of double bonds and cyclopropane units present on all acyl chains or all branches of the corresponding lipid molecule. Because both numbers are crucial to membrane fluidity [54], this analysis can provide valuable insights into their effects on aromatic tolerance.

Phenol is a lignin depolymerization product model compound that contains an aromatic ring and a hydroxyl group. It is present in a variety of depolymerized lignin streams at low concentrations [55–57]. Phenol, like many lignin-derived aromatic compounds, is hypothesized to disrupt cellular membranes due to the hydrophobicity of the aromatic ring [58]. It also has a toxicity level that is similar to many other lignin-derived aromatic compounds [59]. In this work, phenol was chosen as a lignin depolymerization product model compound for studying changes in membrane composition that were hypothesized to be correlated with evolutionary changes that led to improved lignin depolymerization product (i.e., phenol) tolerance and utilization [20]. Increasing lignin depolymerization product tolerance in *R. opacus* could improve biorefinery economics by increasing product titers, yields, and productivities for lignin valorization via a hybrid conversion approach.

To explore the relationship between lipid metabolism and aromatic tolerance in *R. opacus*, we characterized lipids from a phenol-adapted *R. opacus* strain (evol40) and the wild-type (WT) strain that used glucose or phenol as a sole carbon source. evol40 was isolated from a culture that was serially passaged on increasing concentrations of phenol as a sole carbon source, and it demonstrated improved phenol tolerance and utilization in our previous work [20]. We hypothesized that (1) both the WT and evol40 strains would alter its lipid composition when grown using phenol compared to glucose as a sole carbon source, and (2) evol40 would have a different lipid composition from that of the WT strain when grown using phenol. We detected significant compositional changes in mycolic acids and phospholipids between strains during growth using phenol. Overall, this work provides new observations of the lipid metabolism of *R. opacus* during growth on a lignin depolymerization model compound and suggests a role of membrane lipid composition in phenol tolerance.

## Methods

### Cell cultures and lipid extraction

*Rhodococcus opacus* PD630 (DSMZ 44193) was used as the WT strain for comparison to the phenol-adapted *R. opacus* strain evol40, which was generated and characterized in our previous work [20]. *R. opacus* strains were grown in a minimal salts medium that was adjusted to pH = 7.0 using 6 M HCl or 2 M NaOH and filter-sterilized using a 0.22-μm filter [20]. Cells were prepared in the same growth conditions (30 °C, 250 r.p.m.) as RNA-Seq experiments as previously described [20]. Briefly, single colonies from LB plates were grown in 2 mL of minimal salts medium supplemented with 0.3 g/L phenol and 1 g/L ammonium sulfate as carbon and nitrogen sources, respectively, in a 50-mL glass culture tube for 48 h. After 48 h, the cell culture was subcultured in increasingly larger volumes of the 0.3 g/L phenol minimal media to prepare enough cells for the initial inoculum (see [20] for more information). To start the main culture, cells were centrifuged at 3000*g* for 10 min at room temperature, resuspended in a minimal salts medium without carbon or nitrogen sources, and added to 100 mL of minimal salts medium supplemented with 1 g/L glucose (glucose), 0.75 g/L phenol (low phenol), or 1.5 g/L phenol (high phenol) as a sole carbon source and 0.05 g/L ammonium sulfate as the nitrogen source at an initial OD_600_ of 0.3. Cultures were grown to early stationary phase (OD_600_ ~ 1 for all cultures). The WT strain did not show significant growth (< 1 doubling after 48 h) using 1.5 g/L phenol as a sole carbon source in volumes larger than 10 mL, so it was not included in our analysis.

Lipids were extracted from cells using a modified Bligh–Dyer method [60]. Briefly, cells were centrifuged at 4600*g* for 15 min at room temperature, and washed twice with 0.9% NaCl. Cell pellets were resuspended in 1 mL of 0.9% NaCl to an OD_600_ of 50, followed by addition of 2 mL chloroform and 1 mL methanol. Next, cells were briefly vortexed and then sonicated on ice using a Q700 sonicator (Qsonica, LLC) with a 10 mm sonicator probe tip at 20% amplitude using a program of 1 s on and 1 s off for 2.5 min. Following sonication, 1 mL of chloroform, followed by 1 mL of 0.9% NaCl, was added to the mixture to induce phase separation, and the bottom organic phase was removed by glass pipet. The upper aqueous phase was re-extracted twice with 3.8 mL chloroform, and the organic phases were pooled and dried under a nitrogen stream and stored at − 20 °C until further use. After extraction, crude lipid extracts were resuspended in 2:1 chloroform:methanol (v/v) for analysis. 50 μg of butylated hydroxytoluene (BHT) was added after extraction to prevent oxidation of lipids.

### Column fractionation of lipid extracts

The crude lipid extracts were separated by solid phase extraction using aminopropyl cartridges at room temperature following a previously described method [61] with some modifications. Briefly, a Macherey–Nagel Chromabond^®^ NH_2_ column (500 mg) was placed under a vacuum manifold for lipid loading and solvent elution with a flow rate of ~ 0.5 mL/min for all solvents. The column was preconditioned with 5 mL of hexane, and ~ 10 mg of lipids dissolved in 500 μL of chloroform:methanol (2:1 v/v) was loaded onto the column. Next, seven solvents were used to elute lipids from the column (in the following order): 2 mL of ethyl acetate:hexane (15:85 v/v), 3 mL of chloroform:methanol (23:1 v/v), 3 mL of methyl-tert-butyl ether:acetic acid (98:5 v/v), 3 mL of acetone:methanol (9:1.35 v/v), 2 mL of chloroform:methanol (2:1 v/v), 2 mL of chloroform:methanol:3.6 M aqueous ammonium acetate (30:60:8 v/v), and 2 mL of 0.1% (v/v) NH_4_OH in methanol. The fractionated lipid samples were dried under a stream of nitrogen and stored at − 20 °C until analysis.

### Liquid chromatography/mass spectrometry (LC/MS) analysis

Lipid extracts were analyzed using an Agilent 1290 Infinity High Performance liquid chromatography system with a 1290 Infinity Autosampler coupled to an Agilent 6460 Triple Quadrupole mass spectrometer with an electrospray ionization source (ESI/MS). Separation of lipids was achieved by a Phenomenex 150 × 2.1 mm (2.7 μ particle size) Kinetex C-18 column at a flow rate of 300 μL/min at room temperature. The mobile phase contained 10 mM ammonium formate (pH 5.0) in solvent A-acetonitrile–water (60:40, v/v) and solvent B-2-propanol-acetonitrile (90:10, v/v). A gradient elution in the following manner was applied: 68% A, 0–1.5 min; 68–55% A, 1.5–4 min; 55–48% A, 4–5 min; 48–42% A, 5–8 min; 42–34% A, 8–11 min; 34–30% A, 11–14 min; 30–25% A, 14–18 min; 25–3% A, 18–25 min; 3–0% A, 25–30 min; 0% A, 30–35 min; 68% A, 35–40 min. Positive ion mode was used to detect TAG species as [M+NH_4_]^+^ ions while negative ion mode was used to analyze phospholipids and mycolic acid species as [M−H]^−^ ions. 40 μL of sample in 1:1 chloroform/methanol (v/v) was injected. The ESI/MS spectra for phospholipids (elution time, 4–24 min, 600–900 Da mass range), mycolic acids (22–35 min, 500–800 Da mass range), and TAGs (20–40 min, 600–1000 Da mass range) were signal averaged using Agilent MassHunter software. Principal component analysis was performed using the *pca* function in MATLAB 2016b.

### Structural characterization by ESI tandem mass spectrometry (MS^n^)

Both high-resolution (*m*/*Δm* = 100 000 at *m/z* 400) higher-energy collision activation dissociation and low-energy collision-induced dissociation tandem mass spectrometry experiments were conducted on a Thermo Scientific (San Jose, CA) LTQ Orbitrap Velos mass spectrometer with Xcalibur operating system. Lipid extracts were dissolved in 0.5% (v/v) NH_4_OH in methanol and were infused (1.5 μL/min) into the ESI source, where the skimmer was set at ground potential, the electrospray needle was set at 4.0 kV, and the temperature of the heated capillary was 300 °C. The automatic gain control of the ion trap was set to 5 × 10^4^, with a maximum injection time of 50 ms. Helium was used as the buffer and collision gas at a pressure of 1 × 10^−3^ mbar (0.75 mTorr). The MS^n^ experiments were carried out with an optimized relative collision energy ranging from 25 to 45% and with an activation *q* value at 0.25, and the activation time at 10 ms to leave a minimal residual abundance of precursor ion (around 20%). The mass selection window for the precursor ions was set at 1 Da wide to admit the monoisotopic ion to the ion trap for collision-induced dissociation for unit resolution detection in the ion trap or high-resolution accurate mass detection in the Orbitrap mass analyzer. Mass spectra were accumulated in the profile mode, typically for 2–10 min for MS^n^ spectra (*n* = 2, 3, 4).

### Statistical analysis

To compare lipid profiles between different strains or different conditions, the total ion counts of identified lipid species were normalized to the total ion counts of identified peaks in their respective lipid classes. Statistical significance was determined using a one mean, two-tailed Student’s *t* test with a threshold of significance of *P* < 0.05.

## Results and discussion

### High-resolution MS^n^ of the WT lipid extract and creation of a lipid species library

Because the lipids of *R. opacus* have not been extensively studied, we performed high-resolution (MS^n^) analysis on fractionated lipid extracts from the WT strain grown using glucose as a carbon source. A total of 23 phospholipids (11 PIs and 12 PEs), 53 mycolic acids, and 44 TAGs were identified (Tables 1, 2, 3). The PI species consisted of C_15_–C_20_ acyl chains with 0–2 double bonds, and PE species contained C_14_–C_19_ acyl chains with 0–2 double bonds (Table 1). These compositions are similar to those observed for other actinomycete strains such as *M. smegmatis* in which PI and PE are also the most abundant phospholipids [62]. However, we did not observe phosphatidylglycerol (PG) and cardiolipin, which have been observed in other actinomycetes [46, 47]. The mycolic acids contained C_30_–C_42_ meromycolate chains with 0–4 double bonds, and C_12_–C_18_ α-branches with 0–1 double bond (Table 2). These structures are also similar to those found in other *Rhodococcus* species [50, 63]. TAG species had 0–4 double bonds and a combined C_36_–C_57_ acyl chain carbons from three acyl groups (Table 3). To account for changes between strains and growth conditions, a lipid species library was also generated based on the observed acyl chain lengths and double bond numbers (Additional file 2: Table S1).

**Table 1:** Phospholipid species identified in *R. opacus* PD630

Measured *m/z* (Da) [M−H]^−^	Rel. intensity (%)	Theoretical mass (Da)	Deviation (mDa)	Composition	Structures	
					Major	Minor isomer
*Phosphatidylinositol (PI) species*						
819.5026	2.1	819.5029	− 0.31	C42 H76 O13 P	17:1/16:1	
821.5181	30.99	821.5186	− 0.41	C42 H78 O13 P	17:1/16:0	18:1/15:0
823.5336	14.78	823.5342	− 0.58	C42 H80 O13 P	17:0/16:0	
833.5182	3.62	833.5186	− 0.31	C43 H78 O13 P	18:1/16:1	
835.5338	65.67	835.5342	− 0.39	C43 H80 O13 P	18:1/16:0	
837.5491	44.79	837.5499	− 0.73	C43 H82 O13 P	18:0/16:0	
847.5337	2.3	847.5342	− 0.51	C44 H80 O13 P	19:1/16:1	18:1/17:1
849.5493	35.85	849.5499	− 0.5	C44 H82 O13 P	19:1/16:0	
851.5648	100	851.5655	− 0.72	C44 H84 O13 P	19:0/16:0	
863.565	2.17	863.5655	− 0.51	C45 H84 O13 P	19:0/17:1	20:1/16:0
865.5806	3.8	865.5812	− 0.56	C45 H86 O13 P	19:0/17:0	
*Phosphatidylethanolamine (PE) species*						
674.4764	4.09	674.4766	− 0.18	C36 H69 O8 N P	17:1/14:0	16:1/15:0
686.4762	5.57	686.4766	− 0.38	C37 H69 O8 N P	16:1/16:0	
688.492	26.9	688.4923	− 0.24	C37 H71 O8 N P	18:1/14:0	17:1/15:0, 16:1/16:0
700.4921	27.92	700.4923	− 0.19	C38 H71 O8 N P	17:1/16:1	
702.5077	57.4	702.5079	− 0.21	C38 H73 O8 N P	17:1/16:0	18:1/15:0
704.5229	1.33	704.5236	− 0.71	C38 H75 O8 N P	17:0/16:0	18:0/15:0, 19:0/14:0
714.5077	75.12	714.5079	− 0.2	C39 H73 O8 N P	18:1/16:1	
716.5233	100	716.5236	− 0.28	C39 H75 O8 N P	18:1/16:0	
718.5385	3.66	718.5392	− 0.76	C39 H77 O8 N P	18:0/16:0	
728.5233	5.25	728.5236	− 0.25	C40 H75 O8 N P	19:1/16:1	18:1/17:1
730.539	29.91	730.5392	− 0.25	C40 H77 O8 N P	19:0/16:1	
732.5546	8.08	732.5549	− 0.24	C40 H79 O8 N P	19:0/16:0

**Table 2:** Mycolic acid species identified in *R. opacus* PD630

Measured *m/z* (Da) [M−H]^−^	Theoretical mass (Da)	Deviation (mDa)	Composition	Rel. intensity (%)	Structure (meromycolate chain/α-branch)	
					Major structures	Other isomers
677.6450	677.6453	− 0.29	C44 H85 O4	0.6		
679.6606	679.6610	− 0.39	C44 H87 O4	0.82		
687.6656	687.6661	− 0.43	C46 H87 O3	1.34	32:2/14:0; 34:2/12:0	30:2/16:0
691.6606	691.6610	− 0.4	C45 H87 O4	0.7		
693.6762	693.6766	− 0.41	C45 H89 O4	1.05		
701.6813	701.6817	− 0.41	C47 H89 O3	2.22	31:2/16:0; 33:2/14:0	32:2/15:0
707.6917	707.6923	− 0.62	C46 H91 O4	0.72		
713.6814	713.6817	− 0.31	C48 H89 O3	1.98	33:2/15:1; 34:3/14:0	
715.6970	715.6974	− 0.38	C48 H91 O3	9.59	32:2/16:0; 34:2/14:0	33:0/15:0; 35:0/13:0
717.7124	717.7130	− 0.62	C48 H93 O3	0.96		
719.6919	719.6923	− 0.36	C47 H91 O4	0.49		
721.7074	721.7079	− 0.58	C47 H93 O4	1.02		
727.6969	727.6974	− 0.44	C49 H91 O3	3.21	36:3/13:0; 35:3/14:0; 33:2/16:1; 34:3/15:0	34:2/15:1; 33:3/16:0; 35:2/14:1; 32:2/17:1
729.7127	729.7130	− 0.33	C49 H93 O3	10.71	36:2/13:0; 35:2/14:0; 33:2/16:0	32:2/17:0
735.7232	735.7236	− 0.42	C48 H95 O4	0.51		
739.6970	739.6974	− 0.35	C50 H91 O3	0.44	34:3/16:1	36:3/14:1
741.7127	741.7130	− 0.35	C50 H93 O3	15.04	36:3/14:0	34:2/16:1; 34:3/16:0
743.7283	743.7287	− 0.42	C50 H95 O3	29.84	34:2/16:0	36:2/14:0; 35:2/15:0
749.7386	749.7392	− 0.62	C49 H97 O4	0.69		
753.7126	753.7130	− 0.42	C51 H93 O3	0.86		
755.7282	755.7287	− 0.45	C51 H95 O3	19.96	36:3/15:0; 37:3/14:0	35:3/16:0; 35:2/16:1; 34:2/17:1; 38:3/13:0
757.7438	757.7443	− 0.5	C51 H97 O3	24.26	36:2/15:0; 37:2/14:0	34:2/17:0; 38:2/13:0
763.7543	763.7549	− 0.56	C50 H99 O4	0.54		
767.7283	767.7287	− 0.41	C52 H95 O3	4.3	36:3/16:1	38:3/14:1
769.7438	769.7443	− 0.48	C52 H97 O3	60.84	36:3/16:0; 38:3/14:0	37:3/15:0; 36:2/16:1
771.7228	771.7236	− 0.76	C51 H95 O4	0.52		
771.7593	771.7600	− 0.7	C52 H99 O3	44.81	36:2/16:0	38:2/14:0; 37:2/15:0
773.7388	773.7392	− 0.39	C51 H97 O4	0.53		
777.7700	777.7705	− 0.55	C51 H101 O4	0.48		
781.7439	781.7443	− 0.44	C53 H97 O3	4.32	37:3/16:1	38:3/15:1; 36:3/17:1
783.7231	783.7236	− 0.44	C52 H95 O4	0.6		
783.7594	783.7600	− 0.53	C53 H99 O3	52.26	37:3/16:0; 38:3/15:0	39:3/14:0; 36:3/17:0
785.7389	785.7392	− 0.37	C52 H97 O4	1.28	h36:3/16:1	
785.7747	785.7756	− 0.88	C53 H101 O3	18.21	37:2/16:0	37:2/17:0; 38:2/15:0
787.7544	787.7549	− 0.47	C52 H99 O4	0.64	h36:3/16:0	
795.7595	795.7600	− 0.43	C54 H99 O3	12.65	38:3/16:1	
797.7392	797.7392	− 0.02	C53 H97 O4	0.78		
797.7749	797.7756	− 0.7	C54 H101 O3	100	38:3/16:0	39:3/15:0; 40:3/14:0
799.7542	799.7549	− 0.67	C53 H99 O4	1.51	h37:3/16:0	h36:3/17:0; h38:3/15:0
801.7697	801.7705	− 0.88	C53 H101 O4	0.5		
809.7751	809.7756	− 0.54	C55 H101 O3	5.99	38:3/17:1; 39:3/16:1	
811.7543	811.7549	− 0.6	C54 H99 O4	1.77		
811.7906	811.7913	− 0.63	C55 H103 O3	37.52	39:3/16:0	38:3/17:0; 40:3/15:0
813.7698	813.7705	− 0.69	C54 H101 O4	2.09	h38:3/16:0	
823.7907	823.7913	− 0.6	C56 H103 O3	5.21	40:4/16:0; 40:3/16:1	39:3/17:1; 38:3/18:1
825.7699	825.7705	− 0.63	C55 H101 O4	1.03		
825.8063	825.8069	− 0.6	C56 H105 O3	27.68	40:3/16:0	39:3/17:0
827.7857	827.7862	− 0.49	C55 H103 O4	0.96		
837.8064	837.8069	− 0.54	C57 H105 O3	1.42	40:3/17:1; 41:3/16:1	
839.7855	839.7862	− 0.71	C56 H103 O4	1		
839.8219	839.8226	− 0.7	C57 H107 O3	3.51	40:3/17:0; 41:2/16:1	
841.8010	841.8018	− 0.85	C56 H105 O4	0.51		
853.8374	853.8382	− 0.81	C58 H109 O3	0.6	42:3/16:0; 41:3/17:0	40:3/18:0

**Table 3:** Triacylglycerol species identified in *R. opacus* PD630

Measured *m/z* (Da) [M+NH_4_]^+^	Rel. intensity (%)	Theoretical mass (Da)	Deviation (mDa)	Composition
654.5664	0.94	654.5667	− 0.34	C39 H76 O6 N
668.5819	0.49	668.5824	− 0.47	C40 H78 O6 N
710.629	0.82	710.6293	− 0.32	C43 H84 O6 N
724.6447	1.45	724.645	− 0.27	C44 H86 O6 N
738.6603	2.35	738.6606	− 0.36	C45 H88 O6 N
750.6605	0.75	750.6606	− 0.15	C46 H88 O6 N
752.6758	2.48	752.6763	− 0.45	C46 H90 O6 N
764.676	1.42	764.6763	− 0.28	C47 H90 O6 N
766.6915	4.18	766.6919	− 0.44	C47 H92 O6 N
768.707	0.37	768.7076	− 0.52	C47 H94 O6 N
776.6772	0.48	776.6763	0.95	C48 H90 O6 N
778.6919	2.17	778.6919	− 0.03	C48 H92 O6 N
780.7072	5.64	780.7076	− 0.41	C48 H94 O6 N
792.7074	5.75	792.7076	− 0.18	C49 H94 O6 N
794.7229	14.41	794.7232	− 0.36	C49 H96 O6 N
806.7233	11.93	806.7232	0.06	C50 H96 O6 N
808.7385	25.6	808.7389	− 0.37	C50 H98 O6 N
820.7388	35.39	820.7389	− 0.08	C51 H98 O6 N
822.7541	63.2	822.7545	− 0.39	C51 H100 O6 N
834.7544	54.02	834.7545	− 0.11	C52 H100 O6 N
836.7696	79.35	836.7702	− 0.55	C52 H102 O6 N
848.7697	76.36	848.7702	− 0.42	C53 H102 O6 N
850.7851	100	850.7858	− 0.69	C53 H104 O6 N
858.7538	0.38	858.7545	− 0.74	C54 H100 O6 N
860.7711	4.48	860.7702	0.96	C54 H102 O6 N
862.7852	49.71	862.7858	− 0.59	C54 H104 O6 N
864.8006	55.85	864.8015	− 0.83	C54 H106 O6 N
874.7853	4.12	874.7858	− 0.54	C55 H104 O6 N
876.8008	39.32	876.8015	− 0.69	C55 H106 O6 N
878.8162	40.34	878.8171	− 0.96	C55 H108 O6 N
888.8008	2.47	888.8015	− 0.71	C56 H106 O6 N
890.8164	17.49	890.8171	− 0.71	C56 H108 O6 N
892.8319	19.93	892.8328	− 0.91	C56 H110 O6 N
902.8162	2.36	902.8171	− 0.87	C57 H108 O6 N
904.832	17.21	904.8328	− 0.73	C57 H110 O6 N
906.8475	18.74	906.8484	− 0.96	C57 H112 O6 N
916.8319	3.39	916.8328	− 0.82	C58 H110 O6 N
918.8476	11.2	918.8484	− 0.84	C58 H112 O6 N
920.8632	9.19	920.8641	− 0.91	C58 H114 O6 N
930.8476	3.06	930.8484	− 0.81	C59 H112 O6 N
932.8633	9.53	932.8641	− 0.81	C59 H114 O6 N
934.8788	5.38	934.8797	− 0.96	C59 H116 O6 N
944.8631	1.96	944.8641	− 0.96	C60 H114 O6 N
946.8788	3.37	946.8797	− 0.87	C60 H116 O6 N

### LC/MS and principal component analysis of WT and evol40 lipid extracts

**Fig. 2:**
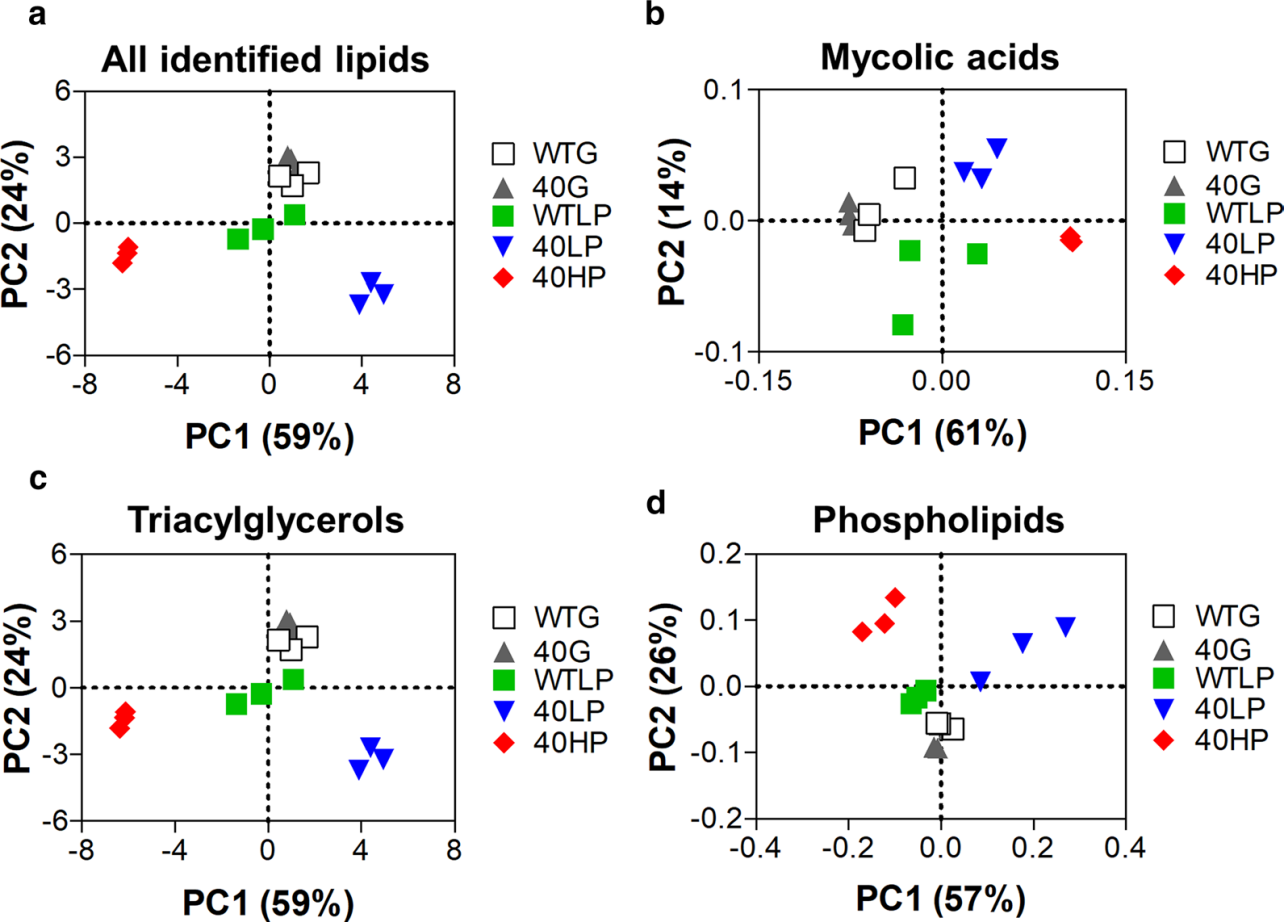
Principal component analysis of lipid types identified using LC/MS. **a** Principal component analysis (PCA) of all identified lipids. **b** PCA of mycolic acid species. **c** PCA of triacylglycerol species. **d** PCA of phospholipid species. Because triacylglycerol ion counts are > 98% of total ion counts in each sample, the PCA plots for all lipid species (**a**) and triacylglycerol species (**c**) are almost identical. Each point represents one replicate. *WTG* WT strain grown in 1 g/L glucose, *40G* evol40 grown in 1 g/L glucose, *WTLP* WT strain grown in 0.75 g/L phenol, *40LP* evol40 strain grown in 0.75 g/L phenol, *40HP* evol40 strain grown in 1.5 g/L phenol, *PC1* 1st principal component, *PC2* 2nd principal component. Percent represents the amount of variance explained by each principal component.

Using LC/MS and our lipid species library, we compared lipid species from the WT and evol40 strains from each growth condition using principal component analysis (PCA) to detect overall changes between samples (Fig. 2; Additional file 2: Table S1): 1 g/L glucose (glucose), 0.75 g/L phenol (low phenol), or 1.5 g/L phenol (high phenol, evol40 only) as a sole carbon source. The WT low phenol, the evol40 low phenol, and the evol40 high phenol data points were generally well-separated from others, while the WT glucose and the evol40 glucose data points clustered together. These results suggest that both strains alter multiple lipid types in low phenol compared to glucose and that the evol40 strain has a lipid profile distinct from that of the WT strain in both phenol growth conditions.

### Profiling of mycolic acid species in different growth conditions

**Fig. 3:**
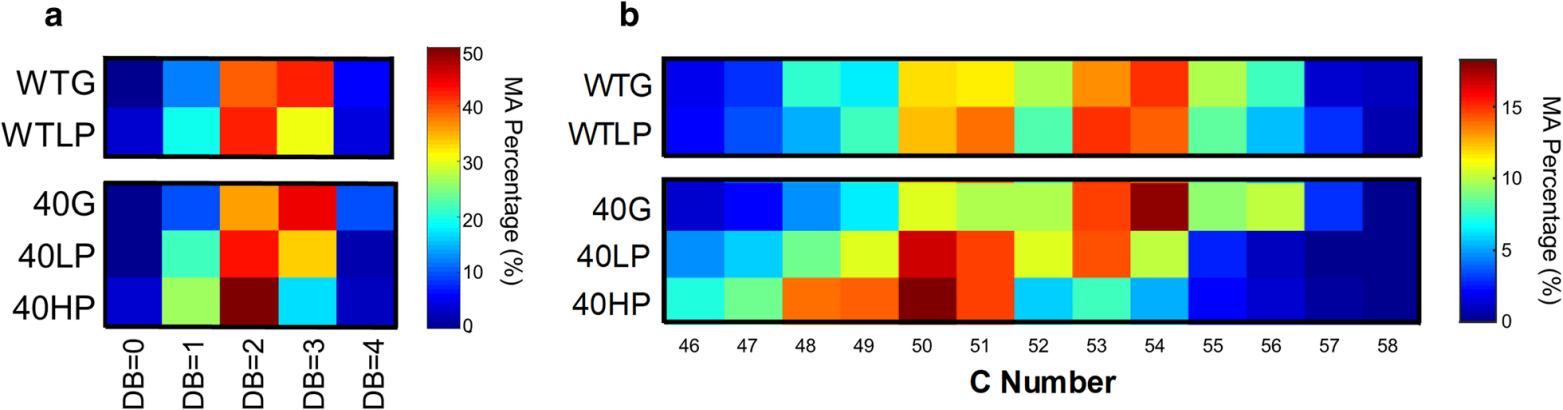
Mycolic acid composition of *R. opacus* strains using glucose or phenol as a sole carbon source. **a** Heat map of double bond (DB) numbers for mycolic acids (MAs) in the WT and evol40 strains. The values shown in the heat map are the average of three replicates. See color bar for scale. The DB number represents the total number of double bonds and cyclopropane units on acyl chains. **b** Heat map of MA carbon (C) number distribution, defined as the total number of carbons located on acyl chains. The values shown in the heat map are the average of three replicates. See color bar for scale. *WTG* WT strain grown in 1 g/L glucose (glucose), *40G* evol40 grown in glucose, *WTLP* WT strain grown in 0.75 g/L phenol (low phenol), *40LP* evol40 strain grown in low phenol, *40HP* evol40 strain grown in 1.5 g/L phenol (high phenol). MA percentage is defined as the total ion counts of each category (DB number or C number) divided by the total ion counts of all mycolic acids detected in each sample.

Mycolic acids have been implicated in the high tolerance of mycobacteria to antibiotics, and they have been shown to change in actinomycetes in response to aromatic compounds [64, 65]. Thus, we hypothesized that WT and evol40 would alter its mycolic acid composition in phenol relative to glucose, and evol40 would have an altered composition compared to the WT strain in phenol. In low phenol relative to glucose, the average mycolic acid double bond number decreased by 12% in the WT strain and by 16% in evol40 (*P* = 0.044 and *P* = 0.003, respectively; one mean, two-tailed Student’s *t* test; Fig. 3a, Additional file 1: Fig. S1, and Additional file 2). However, the average mycolic acid double bond number was not significantly different between the two strains in low phenol (*P* = 0.746). For evol40, this average decreased further by 13% in high phenol relative to low phenol (*P* = 0.008). These observations are consistent with the findings obtained by GC/MS analysis that fatty acids derived from mycolic acids in *R. opacus* PWD4 had decreased double bond numbers during growth in the presence of chlorophenol (not as a sole carbon source) compared to the absence of chlorophenol [52]. Thus, our observations may indicate that increased mycolic acid saturation is a general response of *R. opacus* species to aromatic compounds.

Carbon numbers of mycolic acids were shifted in phenol relative to glucose (Fig. 3b). In the WT strain, the average carbon number of mycolic acids was similar in both glucose and low phenol (*P* = 0.79), while the average carbon number of mycolic acids decreased by ~ 2 carbons (*P* = 0.001) for evol40 in low phenol relative to glucose (Fig. 3b, Additional file 1: Figs. S2, S3). The average mycolic acid carbon number was also significantly different between the two strains in low phenol (*P* = 0.027), suggesting that the two strains remodel their mycomembranes differently in response to phenol. In addition, evol40 decreased its average mycolic acid carbon number by another ~ 1 carbon in high phenol relative to low phenol (*P* = 0.023; Additional file 1: Figs. S2, S3). Decreased mycolic acid chains lengths were also observed for *R. opacus* PWD4 in the presence of chlorophenol [52]. Overall, shorter mycolic acid chain lengths in evol40 during growth using phenol suggest that potential alterations in mycomembrane composition could affect phenol tolerance.

### Profiling of phospholipid species in different growth conditions

**Fig. 4:**
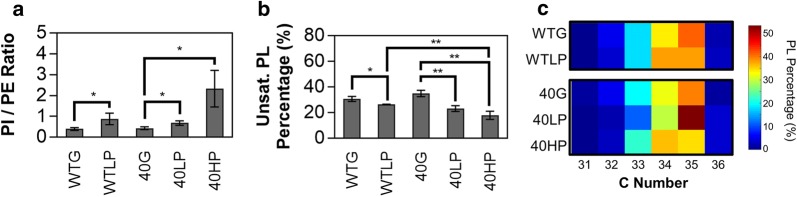
Phospholipid composition of *R. opacus* strains using glucose or phenol as a sole carbon source. **a** Ratio of phosphatidylinositol (PI) to phosphatidylethanolamine (PE) total ion counts in the WT and evol40 strains using glucose or phenol as a sole carbon source. **b** Percentage of unsaturated phospholipids (PLs) (i.e., phospholipids with at least one unsaturated fatty acyl substituent) in WT and evol40 strains using glucose or phenol as a sole carbon source. **c** Distribution of phospholipid carbon (C) numbers in WT and evol40 strains. Here, C number is defined as the total number of acyl chain carbons, and unsaturation means double bonds and cyclic chains on acyl substituents. Each square is the average of three replicates. PL percentage is defined as the total ion counts of each category (unsaturated phospholipids or C number) divided by the total ion counts of all detected PI and PE species in the sample. For A and B, bars represent the average of three replicates, error bars represent one standard deviation, and statistical significance was determined using a one mean, two-tailed Student’s *t* test (*P < 0.05; **P < 0.01). *WTG* WT strain grown in 1 g/L glucose (glucose), *40G* evol40 grown in glucose, *WTLP* WT strain grown in 0.75 g/L phenol (low phenol), *40LP* evol40 strain grown in low phenol, *40HP* evol40 strain grown in 1.5 g/L phenol (high phenol).

The inner membrane consists of phospholipids in *R. opacus* (Fig. 1), and some bacteria modify phospholipid head groups and the number and type of double bonds in response to organic solvents and aromatic compounds [40, 66, 67]. We compared the ratio of the two dominant phospholipid species in *R. opacus*, PI and PE, for each strain and growth condition, hypothesizing that *R. opacus* might also change phospholipid composition during growth using phenol as a sole carbon source (Fig. 4a). The PI/PE ratio increased by 130% for the WT strain and 60% for the evol40 strain in the low phenol condition compared to the glucose condition (*P* = 0.042 and *P* = 0.026, respectively; one mean, two-tailed Student’s *t* test). However, there was not a significant difference in the PI/PE ratio between the WT and the evol40 strain in the low phenol condition (*P* = 0.313). In the evol40 strain, the PI/PE ratio was 240% higher in high phenol relative to low phenol and 440% higher relative to glucose (*P* = 0.032 and *P* = 0.020, respectively; one mean, two-tailed Student’s *t* test). PI is an uncommon phospholipid in bacteria and is more commonly found in eukaryotes such as yeast [68]. In actinomycete strains, PI is an essential phospholipid for growth, and it is a precursor for PI-mannoside and other membrane components such as lipoarabinomannan [62, 69]. Modification of phospholipid head groups has been shown to increase tolerance to various compounds in *Escherichia coli*, including aromatic compounds [70]. This work demonstrates the first study in bacteria where PI is increased in response to phenol and suggests that phospholipid head groups, specifically inositol head groups, could be related to phenol tolerance in *R. opacus*.

Shifts in the average phospholipid double bond number were also observed (Fig. 4b). Both the WT and evol40 strains had lower percentages of unsaturated phospholipids in low phenol relative to glucose, and the evol40 strain had even lower percentages in high phenol relative to low phenol and glucose (Fig. 4b), a trend that was also observed for mycolic acids (Fig. 3a). This trend toward fewer number of unsaturation (double bonds and cyclic chains) was also generally observed for PI and PE separately (Additional file 1: Fig. S4). While reduction in phospholipid unsaturation may improve tolerance to phenol, it could negatively affect respiratory metabolism and growth by reducing the diffusion of enzymes and electron carriers within the membrane [71].

Although we observed significant changes in chain lengths for mycolic acids in evol40 (Fig. 3b), relatively minor shifts in phospholipid chain lengths occurred, and no clear trend emerged between strains or growth conditions (Fig. 4c, Additional file 1: Figs. S4, S5). Studies in other organisms have shown that the addition of phenol to cultures can also decrease the double bond number of phospholipids [40, 66], and studies of derivatized fatty acids from *R. opacus* GM-14, GM-29, and 1CP grown in aromatic compounds also had fewer double bonds compared to those from cells grown using fructose as a sole carbon source [49]. These results suggest that phospholipid unsaturation could be related to phenol tolerance in *R. opacus* species, and modification of phospholipid composition could improve aromatic tolerance and utilization.

### Profiling of triacylglycerol species in different growth conditions

**Fig. 5:**
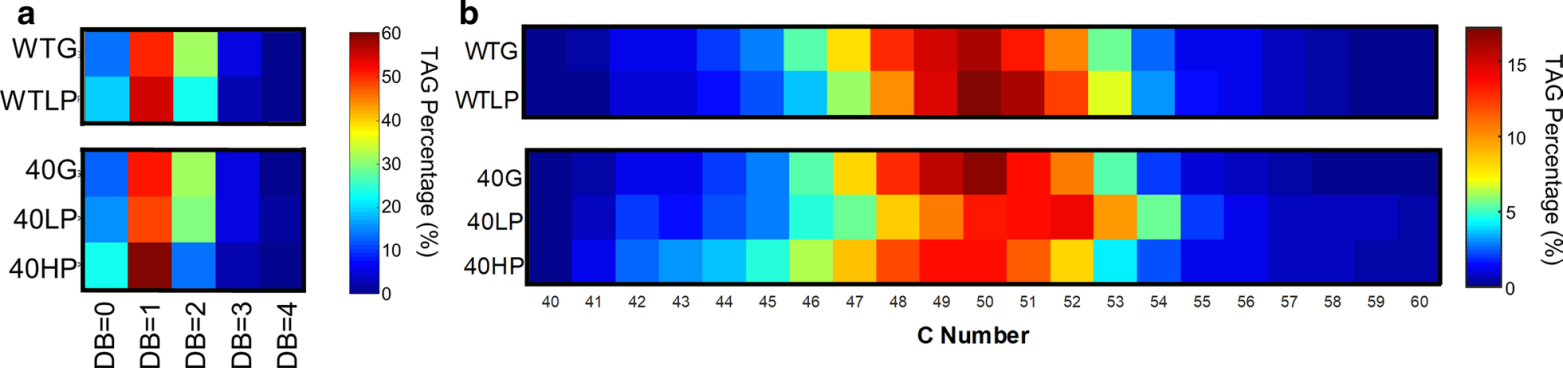
Triacylglycerol compositions of WT and evol40 strains using glucose or phenol as a sole carbon source. **a** Distribution of TAG double bond (DB) numbers in the WT and evol40 strains in each condition. The DB number represents the total number of double bonds and cyclopropane units on acyl chains. **b** Heat map of TAG carbon (C) number distribution, with the C number defined as the total number of acyl chain carbons. TAG percentage is defined as the total ion counts of each category (DB number or C number) divided by the total ion counts of all TAGs detected in each sample. Values in the heat map are the average of three replicates. See color bar for scale. *WTG* WT strain grown in 1 g/L glucose, *40G* evol40 grown in 1 g/L glucose, *WTLP* WT strain grown in 0.75 g/L phenol, *40LP* evol40 strain grown in 0.75 g/L phenol, *40HP* evol40 strain grown in 1.5 g/L phenol.

*R. opacus* accumulates large amounts of TAGs under nitrogen limitation using many different carbon sources [25]. To determine if any changes occurred in TAGs, a lipid class important for biofuel production, we analyzed the TAG composition in each strain and growth condition (Fig. 5). We hypothesized that the TAG profile would be different between glucose and low phenol for both strains and between the WT and evo40 strains in phenol. The WT strain decreased the average TAG double bond number by 14% in low phenol relative to glucose, while the average TAG double bond number remained roughly the same between the two conditions in the evol40 strain (*P* = 0.005 and *P* = 0.93, respectively; one mean, two-tailed Student’s *t* test; Fig. 5a, Additional file 1: Fig. S6). However, evol40 had a 25% lower average TAG double bond number in high phenol relative to low phenol (*P* = 5 × 10^−4^), which was also significantly lower than the WT in low phenol (*P* = 0.016). This shift to fewer double bonds in the WT strain also occurred in *R. opacus* GM-14, GM-29, and 1CP, in which growth in phenol as a sole carbon source reduced the percentage of double bonds on fatty acid chains compared to fructose as a sole carbon source [49]. The average TAG carbon number slightly increased in low phenol for both strains relative to glucose, but it decreased for evol40 in high phenol relative to low phenol, which makes the trend less clear (Fig. 5b, Additional file 1: Figs. S7, S8). These changes, along with changes in the mycolic acid and phospholipid compositions, suggest that WT and evol40 respond differently to phenol, and lipid unsaturation could play a role in phenol tolerance in *R. opacus*.

## Conclusions

Hybrid chemical and biological approaches to lignin valorization require a deeper understanding of bacterial aromatic tolerance and utilization mechanisms. *R. opacus* is a promising host for the biological conversion of lignin-derived aromatic compounds into valuable products, but the role of its lipid metabolism in aromatic tolerance is not well understood. This study characterized the three lipid types (mycolic acids, phospholipids, and TAGs) in the WT and a phenol-adapted strain of *R. opacus* (evol40) when grown using glucose or phenol as a sole carbon source. For all lipid species characterized, a general trend toward fewer double bonds in lipid species was observed during growth using phenol. Mycolic acids had fewer double bonds during growth using phenol compared to glucose for both strains, and mycolic acid chain lengths in evol40, but not in the WT strain, were significantly shorter in phenol conditions compared to the glucose condition, showing the similarity and difference in phenol responses between the two strains. The relative amount of PI increased in phenol growth conditions, which suggests that phospholipid head groups, specifically inositol head groups, could play a role in *R. opacus* phenol tolerance. Overall, this work represents the first lipidomic study of membrane and TAG lipids in *R. opacus* using phenol as a sole carbon source. These results suggest that the lipid metabolism of *R. opacus* is related to phenol tolerance by affecting the mycomembrane and phospholipid membrane compositions during growth using phenol. We envision that the aromatic tolerance of *R. opacus* or other strains of interest can be further understood and engineered for improved lignin hybrid/bioconversion by changing and controlling expression levels of relevant enzymes, including fatty acid desaturases, cyclopropane fatty acid synthases, and phospholipid biosynthesis enzymes.

## Additional files

**Additional file 1: Figure S1.** Double bond (DB) numbers of mycolic acid (MA) species. A. Distribution of mycolic acid DB numbers. B. Average mycolic acid DB numbers. The average DB number was calculated using the following equation: Average DB number = ∑*F*_*i*_*n*_*i*_, where *F* is the fraction of the total mycolic acids with a particular double bond number and *n* is the corresponding double bond number. WTG = WT strain grown in 1 g/L glucose, 40G = evol40 grown in 1 g/L glucose. WTLP = WT strain grown in 0.75 g/L phenol, 40LP = evol40 strain grown in 0.75 g/L phenol, 40HP = evol40 strain grown in 1.5 g/L phenol. MA percentage is defined as the total ion counts of each category (i.e. DB number) divided by the total ion counts of all detected MA species in the sample. Bars represent the average of three replicates, and error bars represent one standard deviation. For A, statistical significance was calculated using a one mean, two tailed Student’s *t* test with *P* < 0.05 as a threshold for statistical significance. Letters above bars indicate statistical significance between strain/growth conditions. For A, a = WTLP vs. 40LP, b = WTLP vs. 40HP, c = 40LP vs. 40HP, d = WTG vs. WTLP, e = 40G vs. 40LP, f = 40G vs. 40HP. For B, *indicates *P* < 0.05, **indicates *P* < 0.01, ***indicates *P* < 0.001. **Figure S2.** Average mycolic acid (MA) carbon (C) numbers. WTG = WT strain grown in 1 g/L glucose, 40G = evol40 grown in 1 g/L glucose. WTLP = WT strain grown in 0.75 g/L phenol, 40LP = evol40 strain grown in 0.75 g/L phenol, 40HP = evol40 strain grown in 1.5 g/L phenol. The average C number was calculated using the following equation: Average C number = ∑*F*_*i*_*C*_*i*_, where *F* is the fraction of the total mycolic acids with a particular carbon number and *C* is the corresponding carbon number. Bars represent the average of three replicates, and error bars represent one standard deviation. Statistical significance was calculated using a one mean, two tailed Student’s *t* test with *P* < 0.05 as a threshold for statistical significance. *P < 0.05, **P < 0.01, ***P < 0.001. **Figure S3.** Carbon (C) number distribution of mycolic acid species. WTG = WT strain grown in 1 g/L glucose, 40G = evol40 grown in 1 g/L glucose. WTLP = WT strain grown in 0.75 g/L phenol, 40LP = evol40 strain grown in 0.75 g/L phenol, 40HP = evol40 strain grown in 1.5 g/L phenol. Mycolic acid percentage is defined as the total ion counts of each category (i.e. C number) divided by the total ion counts of all detected mycolic acid species in the sample. Bars represent the average of three replicates, and error bars represent one standard deviation. **Figure S4.** Phosphatidylinositol (PI) and phosphatidylethanolamine (PE) average carbon (C) number and unsaturation percentage. A. Average PI carbon number. B. Average PE carbon number. C. Percentage of unsaturated PI species. D. Percentage of unsaturated PE species. WTG = WT strain grown in 1 g/L glucose, 40G = evol40 grown in 1 g/L glucose. WTLP = WT strain grown in 0.75 g/L phenol, 40LP = evol40 strain grown in 0.75 g/L phenol, 40HP = evol40 strain grown in 1.5 g/L phenol. The average C number was calculated using the following equation: Average C number = ∑*F*_*i*_*C*_*i*_, where *F* is the fraction of the total PI or PE species with a particular carbon number and *C* is the corresponding carbon number. Unsaturated PI (or PE) percentage is defined as the total ion counts of PI (or PE) species with at least one unsaturated fatty acyl substituent divided by the total ion counts of all detected PI (or PE) species in the sample. Bars represent the average of three replicates, and error bars represent one standard deviation. Statistical significance was calculated using a one mean, two tailed Student’s *t* test with *P* < 0.05 as a threshold for statistical significance. For C and D, *P < 0.05, **P < 0.01, ***P < 0.001. **Figure S5.** Carbon (C) number distribution of phospholipid (PL) species. WTG = WT strain grown in 1 g/L glucose, 40G = evol40 grown in 1 g/L glucose. WTLP = WT strain grown in 0.75 g/L phenol, 40LP = evol40 strain grown in 0.75 g/L phenol, 40HP = evol40 strain grown in 1.5 g/L phenol. PL percentage is defined as the total ion counts of each category (i.e. C number) divided by the total ion counts of all detected PL species in the sample. Bars represent the average of three replicates, and error bars represent one standard deviation. Statistical significance was calculated using a one mean, two tailed Student’s *t* test with *P* < 0.05 as a threshold for statistical significance. Letters above bars indicate statistical significance between strain/growth conditions. a = WTLP vs. 40LP, b = WTLP vs. 40HP, c = 40LP vs. 40HP, d = WTG vs. WTLP, e = 40G vs. 40LP, f = 40G vs. 40HP. **Figure S6.** Triacylglycerol (TAG) double bond (DB) numbers. A. Distribution of TAG double bond (DB) numbers. B. Average TAG DB numbers. The average DB number for each sample was calculated using the following equation: Average DB number = ∑*F*_*i*_*n*_*i*_, where *F* is the fraction of the total TAGs with a particular double bond number and *n* is the corresponding double bond number. WTG = WT strain grown in 1 g/L glucose, 40G = evol40 grown in 1 g/L glucose. WTLP = WT strain grown in 0.75 g/L phenol, 40LP = evol40 strain grown in 0.75 g/L phenol, 40HP = evol40 strain grown in 1.5 g/L phenol. TAG percentage is defined as the total ion counts of each category (i.e. DB number) divided by the total ion counts of all detected TAG species in the sample. Bars represent the average of three replicates, and error bars represent one standard deviation. For A, statistical significance was calculated using a one mean, two tailed Student’s *t* test with *P* < 0.05 as threshold for statistical significance. Letters above bars indicate statistical significance between strain/growth conditions. For A, a = WTLP vs. 40LP, b = WTLP vs. 40HP, c = 40LP vs. 40HP, d = WTG vs. WTLP, e = 40G vs. 40LP, f = 40G vs. 40HP. For B, *P < 0.05, **P < 0.01, ***P < 0.001. **Figure S7.** Triacylglycerol (TAG) carbon (C) number distribution. WTG = WT strain grown in 1 g/L glucose, 40G = evol40 grown in 1 g/L glucose. WTLP = WT strain grown in 0.75 g/L phenol, 40LP = evol40 strain grown in 0.75 g/L phenol, 40HP = evol40 strain grown in 1.5 g/L phenol. TAG percentage is defined as the total ion counts of each category (i.e. C number) divided by the total ion counts of all detected TAG species in the sample. Bars represent the average of three replicates, and error bars represent one standard deviation. **Figure S8.** Average triacylglycerol (TAG) carbon (C) number by strain and growth condition. WTG = WT strain grown in 1 g/L glucose, 40G = evol40 grown in 1 g/L glucose. WTLP = WT strain grown in 0.75 g/L phenol, 40LP = evol40 strain grown in 0.75 g/L phenol, 40HP = evol40 strain grown in 1.5 g/L phenol. The average C number for each sample was calculated using the following equation: Average C number = ∑*F*_*i*_*C*_*i*_, where *F* is the fraction of the total TAGs with a particular carbon number and *C* is the corresponding carbon number. Bars represent the average of three replicates, and error bars represent one standard deviation. Statistical significance was calculated using a one mean, two tailed Student’s *t* test with *P* < 0.05 as threshold for statistical significance. *P < 0.05, **P < 0.01, ***P < 0.001.

**Additional file 2: Table S1.** Excel file containing the lipid library and lipid library matches from LC/MS data. For all tabs, WTG = WT strain grown in 1 g/L glucose (glucose), 40G = evol40 grown in glucose, WTLP = WT strain grown in 0.75 g/L phenol (low phenol), 40LP = evol40 strain grown in low phenol, 40HP = evol40 strain grown in 1.5 g/L phenol (high phenol), and SD = standard deviation. Lipid species are abbreviated using their carbon number (C) and double bond number (DB), where the carbon number is the number of acyl carbons and the double bond number is the number of double bonds and cyclopropane units on acyl chains. *Tab 1. TAG library.* Library of triacylglycerol (TAG) species based on MS^n^ data. Lipid species in the library that were observed in MS^n^ analysis (Observed MS^n^ column) are indicated using “x” (see Table 3). *Tab 2. TAG data.* Normalized TAG species ion counts from LC/MS data. Species amounts in each replicate (rep) are normalized by dividing the total ion counts of individual species by the total ion counts of all detected TAG species in the sample. *Tab 3. PL library.* Library of phospholipid (PL) species based on MS^n^ data. Lipid species in the library that were observed in MS^n^ analysis (Observed MS^n^ column) are indicated using “x” (see Table 1). *Tab 4. PL data.* Normalized PL species ion counts from LC/MS data. Species amounts in each replicate (rep) are normalized by dividing the total ion counts of individual species by the total ion counts of all detected phosphatidylinositol (PI) or phosphatidylethanolamine (PE) species in the sample. *Tab 5. MA library.* Library of mycolic acid (MA) species based on MS^n^ data. Lipid species in the library that were observed in MS^n^ analysis (Observed MS^n^ column) are indicated using “x” (see Table 2). *Tab 6. MA data.* Normalized MA species ion counts from LC/MS data. Species amounts in each replicate (rep) are normalized by dividing the total ion counts of individual species by the total ion counts of all detected MA species in the sample. *Tab 7. Statistical tests. P* values for statistical tests comparing C number and DB number for each lipid class between strains or growth conditions. Statistical significance was determined using a one mean, two-tailed Student’s *t* test with a threshold of significance of *P* < 0.05.
