# Supplementary material for: Lipid metabolism of phenol-tolerant Rhodococcus opacus strains for lignin bioconversion
Source: Biotechnol Biofuels. 2018 Dec 28;11:339. doi: 10.1186/s13068-018-1337-z (PMC6309088; doi:10.1186/s13068-018-1337-z)
Supplement: Supplementary file 1 — Additional file 1: Figure S1. Double bond (DB) numbers of mycolic acid (MA) species. A. Distribution of mycolic acid DB numbers. B. Average mycolic acid DB numbers. The average DB number was calculated using the following equation: Average DB number = ∑Fini, where F is the fraction of the total mycolic acids with a particular double bond number and n is the corresponding double bond number. WTG = WT strain grown in 1 g/L glucose, 40G = evol40 grown in 1 g/L glucose. WTLP = WT strain grown in 0.75 g/L phenol, 40LP = evol40 strain grown in 0.75 g/L phenol, 40HP = evol40 strain grown in 1.5 g/L phenol. MA percentage is defined as the total ion counts of each category (i.e. DB number) divided by the total ion counts of all detected MA species in the sample. Bars represent the average of three replicates, and error bars represent one standard deviation. For A, statistical significance was calculated using a one mean, two tailed Student’s t test with P < 0.05 as a threshold for statistical significance. Letters above bars indicate statistical significance between strain/growth conditions. For A, a = WTLP vs. 40LP, b = WTLP vs. 40HP, c = 40LP vs. 40HP, d = WTG vs. WTLP, e = 40G vs. 40LP, f = 40G vs. 40HP. For B, *indicates P < 0.05, **indicates P < 0.01, ***indicates P < 0.001. Figure S2. Average mycolic acid (MA) carbon (C) numbers. WTG = WT strain grown in 1 g/L glucose, 40G = evol40 grown in 1 g/L glucose. WTLP = WT strain grown in 0.75 g/L phenol, 40LP = evol40 strain grown in 0.75 g/L phenol, 40HP = evol40 strain grown in 1.5 g/L phenol. The average C number was calculated using the following equation: Average C number = ∑FiCi, where F is the fraction of the total mycolic acids with a particular carbon number and C is the corresponding carbon number. Bars represent the average of three replicates, and error bars represent one standard deviation. Statistical significance was calculated using a one mean, two tailed Student’s t test with P < 0.05 as a threshold for stati [file 13068_2018_1337_MOESM1_ESM.pdf]

Additional file 1

Lipid metabolism of phenol tolerant *Rhodococcus opacus* strains for lignin bioconversion

William R. Henson, Fong Fu Hsu, Gautam Dantas, Tae Seok Moon, Marcus Foston

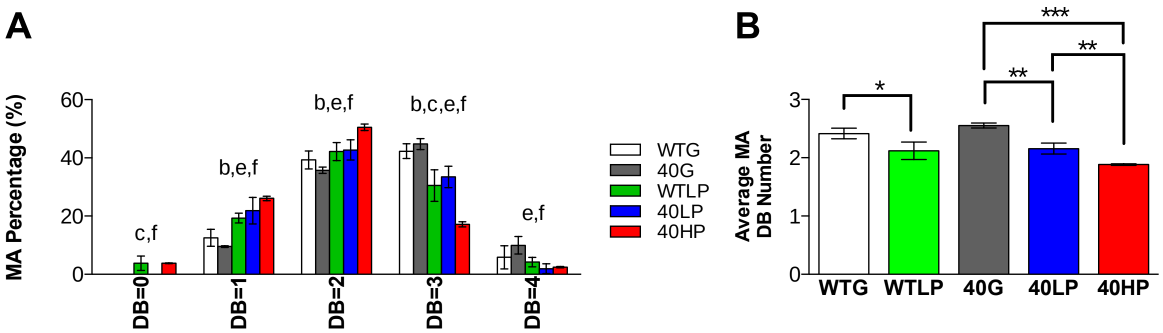

Figure S1. Double bond (DB) numbers of mycolic acid (MA) species. A. Distribution of mycolic acid DB numbers. B. Average mycolic acid DB numbers. The average DB number was calculated using the following equation: $Average\ DB\ number = \sum F_i n_i$, where F is the fraction of the total mycolic acids with a particular double bond number and n is the corresponding double bond number. WTG = WT strain grown in 1 g/L glucose, 40G = evol40 grown in 1 g/L glucose. WTLP = WT strain grown in 0.75 g/L phenol, 40LP = evol40 strain grown in 0.75 g/L phenol, 40HP = evol40 strain grown in 1.5 g/L phenol. MA percentage is defined as the total ion counts of each category (i.e. DB number) divided by the total ion counts of all detected MA species in the sample. Bars represent the average of three replicates, and error bars represent one standard deviation. For A, statistical significance was calculated using a one mean, two tailed Student's t-test with P < 0.05 as a threshold for statistical significance. Letters above bars indicate statistical significance between strain/growth conditions. For A, a = WTLP vs. 40LP, b = WTLP vs. 40HP, c = 40LP vs. 40HP, d = WTG vs. WTLP, e = 40G vs. 40LP, f = 40G vs. 40HP. For B, * indicates P < 0.05, ** indicates P < 0.01, *** indicates P < 0.001.

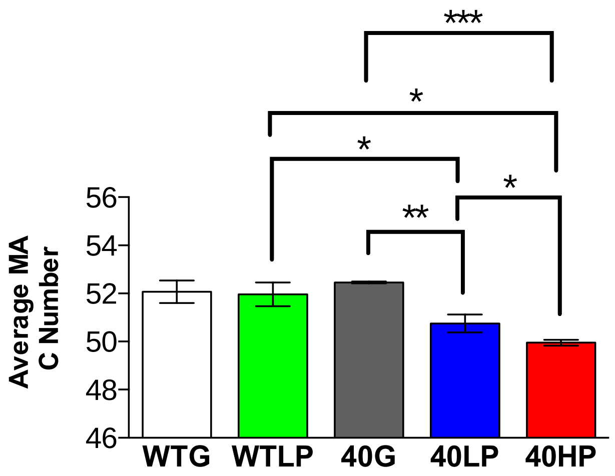

Figure S2. Average mycolic acid (MA) carbon (C) numbers. WTG = WT strain grown in 1 g/L glucose, 40G = evol40 grown in 1 g/L glucose. WTLP = WT strain grown in 0.75 g/L phenol, 40LP = evol40 strain grown in 0.75 g/L phenol, 40HP = evol40 strain grown in 1.5 g/L phenol. The average C number was calculated using the following equation: $Average\ C\ number = \sum F_i C_i$, where F is the fraction of the total mycolic acids with a particular carbon number and C is the corresponding carbon number. Bars represent the average of three replicates, and error bars represent one standard deviation. Statistical significance was calculated using a one mean, two tailed Student's t-test with $P < 0.05$ as a threshold for statistical significance. * indicates $P < 0.05$, ** indicates $P < 0.01$, *** indicates $P < 0.001$.

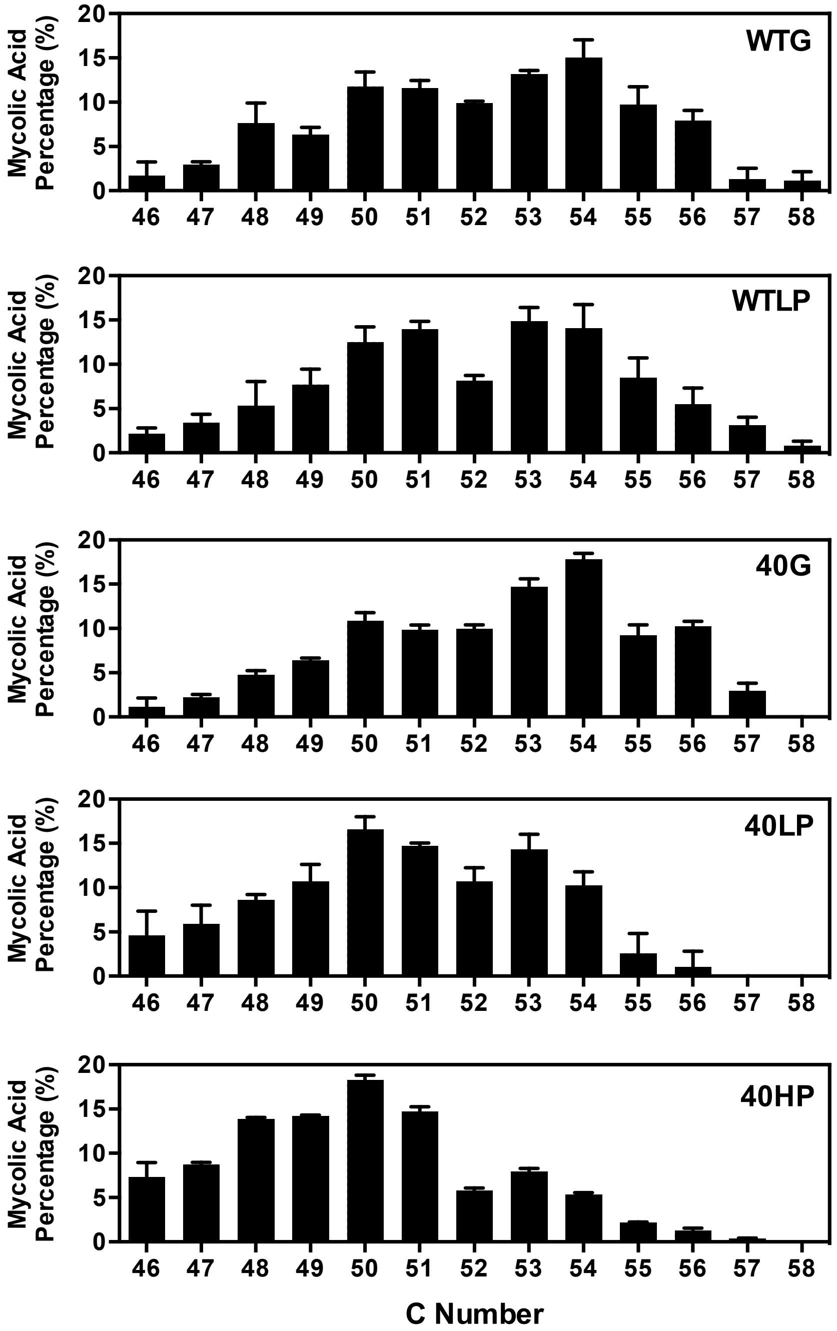

Figure S3. Carbon (C) number distribution of mycolic acid species. WTG = WT strain grown in 1 g/L glucose, 40G = evol40 grown in 1 g/L glucose. WTLP = WT strain grown in 0.75 g/L phenol, 40LP = evol40 strain grown in 0.75 g/L phenol, 40HP = evol40 strain grown in 1.5 g/L phenol. Mycolic acid percentage is defined as the total ion counts of each category (i.e. C number) divided by the total ion counts of all detected mycolic acid species in the sample. Bars represent the average of three replicates, and error bars represent one standard deviation.

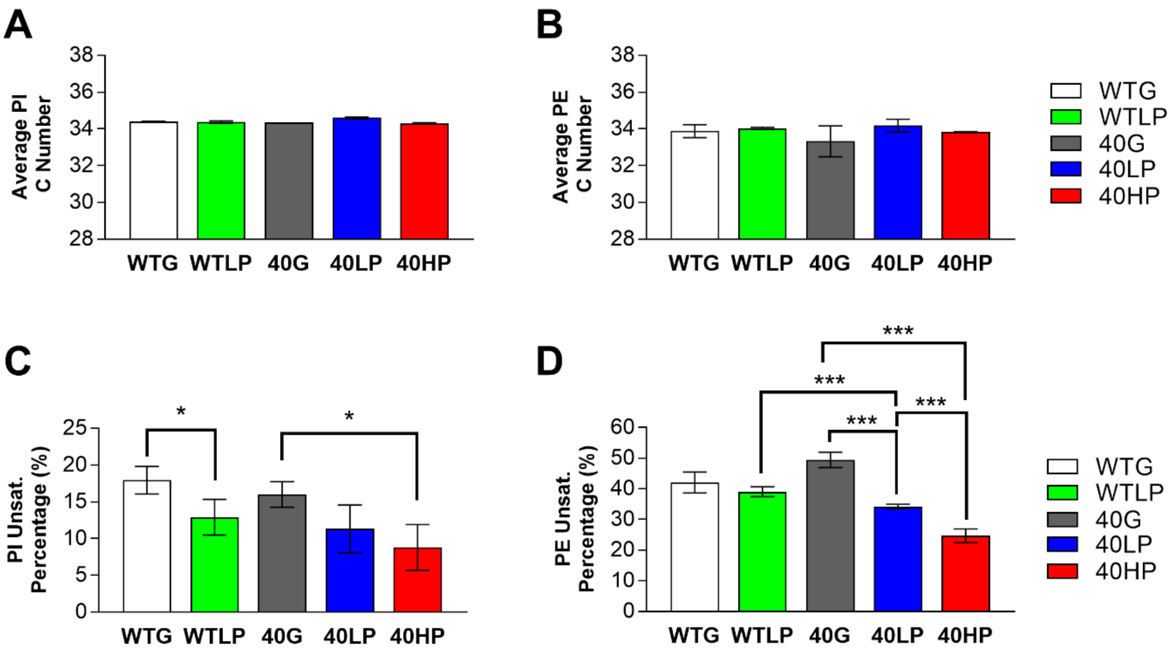

Figure S4. Phosphatidylinositol (PI) and phosphatidylethanolamine (PE) average carbon (C) number and unsaturation percentage. A. Average PI carbon number. B. Average PE carbon number. C. Percentage of unsaturated PI species. D. Percentage of unsaturated PE species. WTG = WT strain grown in 1 g/L glucose, 40G = evol40 grown in 1 g/L glucose. WTLP = WT strain grown in 0.75 g/L phenol, 40LP = evol40 strain grown in 0.75 g/L phenol, 40HP = evol40 strain grown in 1.5 g/L phenol. The average C number was calculated using the following equation: $Average\ C\ number = \sum F_i C_i$, where F is the fraction of the total PI or PE species with a particular carbon number and C is the corresponding carbon number. Unsaturated PI (or PE) percentage is defined as the total ion counts of PI (or PE) species with at least one unsaturated fatty acyl substituent divided by the total ion counts of all detected PI (or PE) species in the sample. Bars represent the average of three replicates, and error bars represent one standard deviation. Statistical significance was calculated using a one mean, two tailed Student's t-test with P < 0.05 as a threshold for statistical significance. For C and D, * indicates P < 0.05, ** indicates P < 0.01, *** indicates P < 0.001.

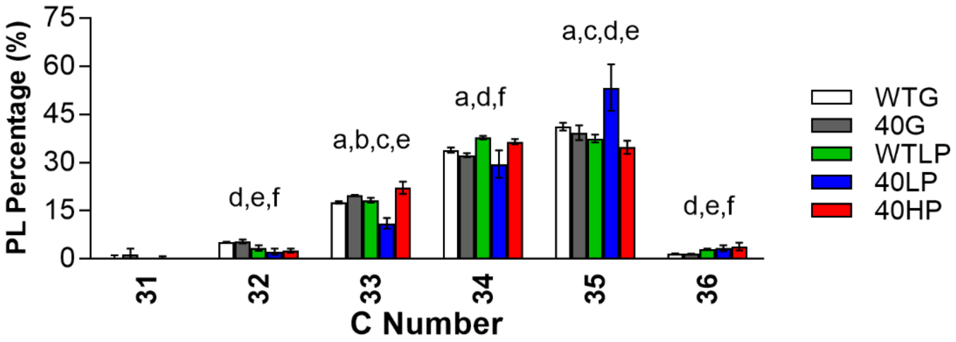

Figure S5. Carbon (C) number distribution of phospholipid (PL) species. WTG = WT strain grown in 1 g/L glucose, 40G = evol40 grown in 1 g/L glucose. WTLP = WT strain grown in 0.75 g/L phenol, 40LP = evol40 strain grown in 0.75 g/L phenol, 40HP = evol40 strain grown in 1.5 g/L phenol. PL percentage is defined as the total ion counts of each category (i.e. C number) divided by the total ion counts of all detected PL species in the sample. Bars represent the average of three replicates, and error bars represent one standard deviation. Statistical significance was calculated using a one mean, two tailed Student's t-test with $P < 0.05$ as a threshold for statistical significance. Letters above bars indicate statistical significance between strain/growth conditions. a = WTLP vs. 40LP, b = WTLP vs. 40HP, c = 40LP vs. 40HP, d = WTG vs. WTLP, e = 40G vs. 40LP, f = 40G vs. 40HP.

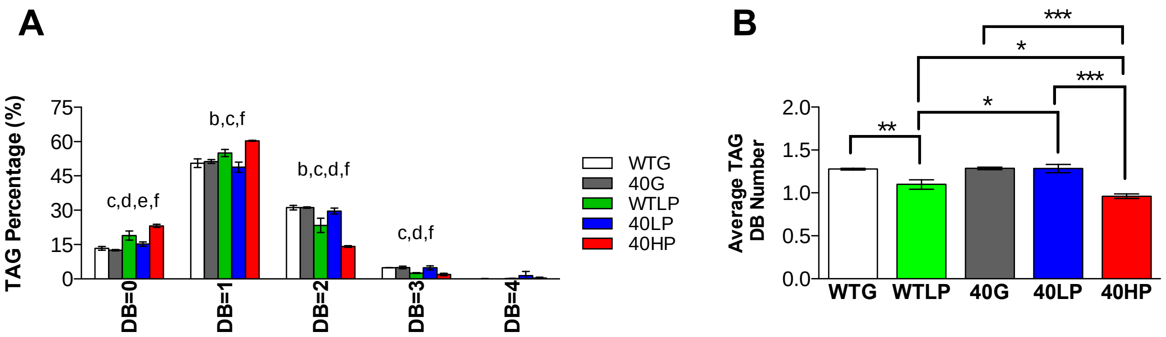

Figure S6. Triacylglycerol (TAG) double bond (DB) numbers. A. Distribution of TAG double bond (DB) numbers. B. Average TAG DB numbers. The average DB number for each sample was calculated using the following equation: $Average\ DB\ number = \sum\ F_i n_i$, where F is the fraction of the total TAGs with a particular double bond number and n is the corresponding double bond number. WTG = WT strain grown in 1 g/L glucose, 40G = evol40 grown in 1 g/L glucose. WTLP = WT strain grown in 0.75 g/L phenol, 40LP = evol40 strain grown in 0.75 g/L phenol, 40HP = evol40 strain grown in 1.5 g/L phenol. TAG percentage is defined as the total ion counts of each category (i.e. DB number) divided by the total ion counts of all detected TAG species in the sample. Bars represent the average of three replicates, and error bars represent one standard deviation. For A, statistical significance was calculated using a one mean, two tailed Student's t-test with P < 0.05 as threshold for statistical significance. Letters above bars indicate statistical significance between strain/growth conditions. For A, a = WTLP vs. 40LP, b = WTLP vs. 40HP, c = 40LP vs. 40HP, d = WTG vs. WTLP, e = 40G vs. 40LP, f = 40G vs. 40HP. For B, * indicates P < 0.05, ** indicates P < 0.01, *** indicates P < 0.001.

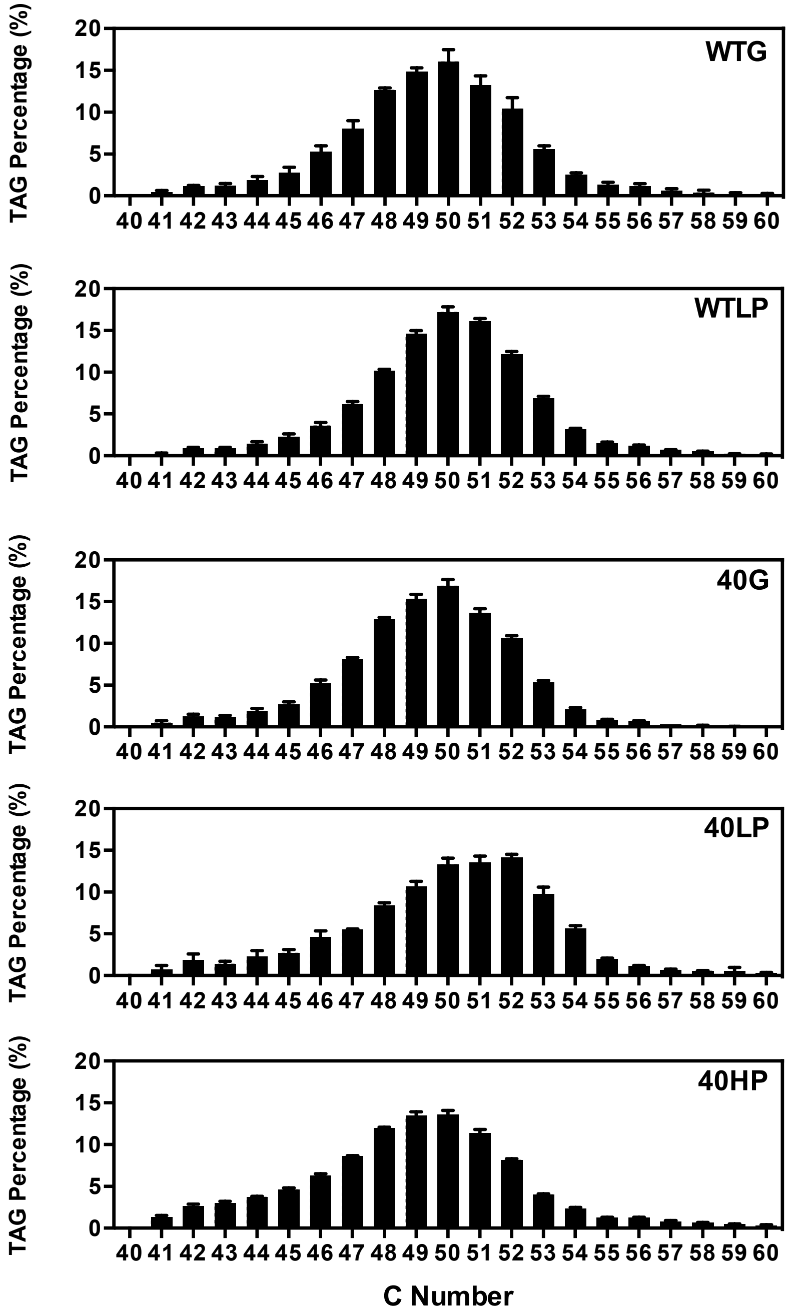

Figure S7. Triacylglycerol (TAG) carbon (C) number distribution. WTG = WT strain grown in 1 g/L glucose, 40G = evol40 grown in 1 g/L glucose. WTLP = WT strain grown in 0.75 g/L phenol, 40LP = evol40 strain grown in 0.75 g/L phenol, 40HP = evol40 strain grown in 1.5 g/L phenol. TAG percentage is defined as the total ion counts of each category (i.e. C number) divided by the total ion counts of all detected TAG species in the sample. Bars represent the average of three replicates, and error bars represent one standard deviation.

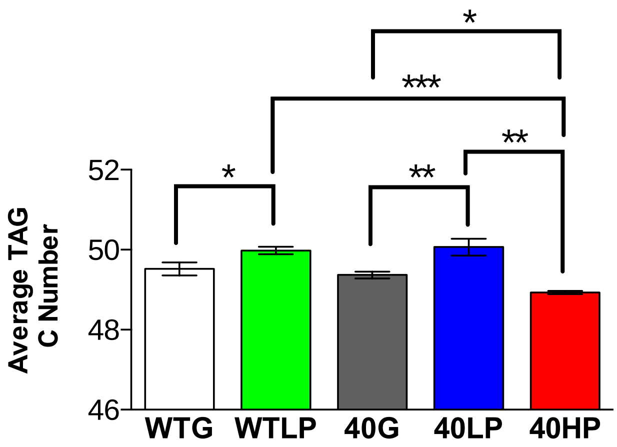

Figure S8. Average triacylglycerol (TAG) carbon (C) number by strain and growth condition. WTG = WT strain grown in 1 g/L glucose, 40G = evol40 grown in 1 g/L glucose. WTLP = WT strain grown in 0.75 g/L phenol, 40LP = evol40 strain grown in 0.75 g/L phenol, 40HP = evol40 strain grown in 1.5 g/L phenol. The average C number for each sample was calculated using the following equation: $Average\ C\ number = \sum\ F_i C_i$, where F is the fraction of the total TAGs with a particular carbon number and C is the corresponding carbon number. Bars represent the average of three replicates, and error bars represent one standard deviation. Statistical significance was calculated using a one mean, two tailed Student's t-test with P < 0.05 as threshold for statistical significance. * indicates P < 0.05, ** indicates P < 0.01, *** indicates P < 0.001.

Table S1. Excel file containing the lipid library and lipid library matches from LC/MS data. For all tabs, WTG = WT strain grown in 1 g/L glucose (glucose), 40G = evol40 grown in glucose, WTLP = WT strain grown in 0.75 g/L phenol (low phenol), 40LP = evol40 strain grown in low phenol, 40HP = evol40 strain grown in 1.5 g/L phenol (high phenol), and SD = standard deviation. Lipid species are abbreviated using their carbon number (C) and double bond number (DB), where the carbon number is the number of acyl carbons and the double bond number is the number of double bonds and cyclopropane units on acyl chains.

Tab 1. TAG library. **Library of triacylglycerol (TAG) species based on MS^n data.** Lipid species in the library that were observed in MS^n analysis (Observed MS^n column) are indicated using “x” (see Table 3).

Tab 2. TAG data. **Normalized TAG species ion counts from LC/MS data.** Species amounts in each replicate (rep) are normalized by dividing the total ion counts of individual species by the total ion counts of all detected TAG species in the sample.

Tab 3. PL library. **Library of phospholipid (PL) species based on MS^n data.** Lipid species in the library that were observed in MS^n analysis (Observed MS^n column) are indicated using “x” (see Table 1).

Tab 4. PL data. **Normalized PL species ion counts from LC/MS data.** Species amounts in each replicate (rep) are normalized by dividing the total ion counts of individual species by the total ion counts of all detected phosphatidylinositol (PI) or phosphatidylethanolamine (PE) species in the sample.

Tab 5. MA library. **Library of mycolic acid (MA) species based on MS^n data.** Lipid species in the library that were observed in MS^n analysis (Observed MS^n column) are indicated using “x” (see Table 2).

Tab 6. MA data. **Normalized MA species ion counts from LC/MS data.** Species amounts in each replicate (rep) are normalized by dividing the total ion counts of individual species by the total ion counts of all detected MA species in the sample.

Tab 7. Statistical tests. **P values for statistical tests comparing C number and DB number for each lipid class between strains or growth conditions.** Statistical significance was determined using a one mean, two-tailed Student’s t-test with a threshold of significance of $P < 0.05$.
